# Supplementary material for: MetaRibo-Seq measures translation in microbiomes
Source: Nat Commun. 2020 Jun 29;11:3268. doi: 10.1038/s41467-020-17081-z (PMC7324362; doi:10.1038/s41467-020-17081-z)
Supplement: Supplementary file 10 — Supplementary Data 7 [file 41467_2020_17081_MOESM10_ESM.zip › File2/Confidence_High_Taxonomy/306812_out.krona.html]

Javascript must be enabled to view this page.

members
magnitude
magnitudeUnassigned
count
unassigned
taxon
rank

306812\_out

2


SRS019029\_contig\_number\_contig-100\_10354.10354
1

superkingdom
1
2

1
phylum
508458

class
1
649775

649776
order
1

649777
family
1

1434006
1
genus

651822

SRS013663\_contig\_number\_contig-100\_47.34357
1
species
